# Microbial Consortium HJ-SH with Very High Degradation Efficiency of Phenanthrene

**DOI:** 10.3390/microorganisms11102383

**Published:** 2023-09-23

**Authors:** Rui Chen, Zhenhua Zhao, Tao Xu, Xiaoqiang Jia

**Affiliations:** 1 Department of Biochemical Engineering, School of Chemical Engineering and Technology, Tianjin University, Tianjin 300072, China; ruichen98@tju.edu.cn (R.C.); zzh197010@126.com (Z.Z.); xt16622899535@163.com (T.X.); 2 Frontier Science Center for Synthetic Biology and Key Laboratory of Systems Bioengineering (MOE), School of Chemical Engineering and Technology, Tianjin University, Tianjin 300350, China; 3 Collaborative Innovation Center of Chemical Science and Engineering (Tianjin), Tianjin University, Tianjin 300072, China

**Keywords:** microbial consortium, PAHs, biodegradation, phenanthrene, tolerance, bioremediation

## Abstract

Phenanthrene (PHE) is one of the model compounds of polycyclic aromatic hydrocarbons (PAHs). In this study, a natural PHE-degrading microbial consortium, named HJ-SH, with very high degradation efficiency was isolated from soil exposed to long-term PHE contamination. The results of GC analysis showed that the consortium HJ-SH degraded 98% of 100 mg/L PHE in 3 days and 93% of 1000 mg/L PHE in 5 days, an efficiency higher than that of any other natural consortia, and even most of the engineered strains and consortia reported so far. Seven dominating strains were isolated from the microbial consortium HJ-SH, named SH-1 to SH-7, which were identified according to morphological observation and 16S rDNA sequencing as *Pseudomonas* sp., *Stenotrophomonas* sp., *Delftia* sp., *Pseudomonas* sp., *Brevundimonas* sp., *Curtobacterium* sp., and *Microbacterium* sp., respectively. Among all the seven single strains, SH-4 showed the strongest PHE degradation ability, and had the biggest degradation contribution. However, it is very interesting that the microbial consortium can hold its high degradation ability only with the co-existence of all these seven single strains. Moreover, HJ-SH exhibited a very high tolerance for PHE, up to 4.5 g/L, and it can degrade some other typical organic pollutants such as biphenyl, anthracene, and n-hexadecane with the degradation ratios of 93%, 92% and 70%, respectively, under 100 mg/L initial concentration in 5 days. Then, we constructed an artificial consortium HJ-7 consisting of the seven single strains, SH-1 to SH-7. After comparing the degradation ratios, cell growth, and relative degradation rates, it was concluded that the artificial consortium HJ-7 with easier reproducibility, better application stability, and larger room for modification can largely replace the natural consortium HJ-SH. In conclusion, this research provided novel tools and new insights for the bioremediation of PHE and other typical organic pollutants using microbial consortia.

## 1. Introduction

Polycyclic aromatic hydrocarbons (PAHs) are semi-volatile and highly polluting organic compounds in the environment [1,2]. PAHs are principally composed of two or more benzene rings [3], arranged in linear, angular, or clustered arrangements [4,5]. A higher number of benzene rings in the structure of PAHs [6] increases their hydrophobicity [7], toxicity, and environmental persistence, while reducing their bioavailability and biodegradability at the same time [8,9]. Anthropogenic factors are the primary sources of PAHs in the environment [10], mainly due to the incomplete combustion of organic materials [11] in industrial processes [12], anaerobic combustion, waste incineration, or landfills [13]. PAHs mixtures have been detected in various environments, and they harm ecosystems and human health due to their toxicity [14], mutagenicity, and carcinogenicity [15]. PAHs such as biphenyl and anthracene are highly toxic to aquatic organisms, and their toxicity is often magnified by metabolism and photo-oxidation in the presence of ultraviolet light [16]. Consequently, developing safe and effective treatment technologies to remediate PAHs pollution in the environment has garnered particular attention [17].

Sixteen PAHs, including naphthalene, acenaphthene, dihydro acenaphthene, fluorene, PHE, anthracene, fluoranthene, pyrene, benzo[a]anthracene, benzo[b]fluoranthene, benzo[k]fluoranthene, benzo[a]pyrene, dibenzo[a, h]anthracene, benzo[g, h, i]perylene and indene[1, 2, 3-c, d]pyrene are listed as priority pollutants by the US Environmental Protection Agency (EPA) [18]. As one of the model compounds of PAHs, PHE consists of three benzene rings, the smallest characteristic unit of carcinogenic PAHs [19]. PHE has low bioavailability and high recalcitrance [20]. Thus, it must be decomposed before being released into the environment [21]. Remediation strategies for PHE-contaminated soils include physical, chemical, and biological methods such as volatilization [22] and chemical precipitation [23]. Among these methods, biological processes are considered the most powerful and effective remediation strategies due to low cost, low energy consumption, and no secondary pollution [24]. Microorganisms can oxidize PHE, break the aromatic structures, and even fully mineralize it [25]. Bioremediation is a biological process used to treat organic pollutants from various media, such as water, soil, air, and the surface of materials. In bioremediation, microorganisms or enzymes are applied to treat and remove organic pollutants from various media [26,27].

PHE-degrading bacteria have been isolated from a wide variety of environments [28], including petroleum-contaminated frozen soils [29], soils of oil refineries [30], and hydrocarbon-impacted sediments [31]. Several *Pseudomonas* sp. have been reported to degrade hydrocarbons with various chain lengths and structures [32]. *P. aeruginosa* W10 was found to degrade PHE [33]. Degradation by microbial consortia is more effective [34], as degradation of contaminant molecules occurs through the cooperative actions of several microbes [35]. At present, the microorganisms capable of degrading PHE, biodegradation pathways of PHE, and their related genes have been reported in detail [19,36,37,38], but the degradation efficiency of most natural and engineered microorganisms is relatively low due to the low bioavailability and high-resistance ability of PHE. It is rather important to search for microorganisms with high PHE-degrading efficiency, providing alternative bacterial agents for the field application of PAHs remediation.

In this study, a natural PHE-degrading microbial consortium HJ-SH with very high degradation efficiency was isolated from PHE-contaminated soil via multiple rounds of domestication and screening. Seven single-strains were isolated and identified, and the degradation ratios of PHE by these strains was calculated via GC analysis. The PHE tolerance threshold of the natural microbial consortium HJ-SH and the degradation contribution of single-strains were researched. And the degradation ability of the consortium HJ-SH on other organic pollutants in the environment was tested. Additionally, the artificial consortium HJ-7 with easier reproducibility, better application stability, and larger room for modification was constructed, which facilitated greater application potential and sustainability for PHE bioremediation strategies. Our study provides a natural consortium with high degradation efficiency as well as its substitutable artificial consortium, which are novel tools to realize efficient degradation and in situ remediation of PHE contamination in the environment.

## 2. Materials and Methods

### 2.1. Chemicals and Culture Media

PAHs (PHE, biphenyl, and naphthalene) and n-hexadecane were purchased from Aladdin (Shanghai, China). The long-term PHE-contaminated soil was obtained from Dagang Oilfield, which was collected from six sampling sites, 100 g for each site (Tianjin, China). Chemical solvents including HPLC-grade dichloromethane, n-hexane, and culture media constituents were purchased from AOBOX (Beijing, China). Reagents and enzymes for polymerase chain reaction (PCR) were provided by Tianjin Guangfu Chemical Reagent Company (Tianjin, China). All chemicals and reagents utilized in this study were of analytical grade (purity ≥ 99%). Beef extract Peptone Medium (BPM) contained 3 g/L beef extract, 10 g/L peptone, 5 g/L NaCl; Yeast extract Peptone Dextrose (YPD) medium contained 10 g/L yeast extract, 20 g/L peptone, 20 g/L glucose; Luria-Bertani (LB) medium contained 5 g/L yeast extract, 10 g/L peptone, and 10 g/L NaCl; Mineral Salt Medium (MSM) contained 5 g/L NaCl, 1 g/L NH_4_NO_3_, 1 g/L K_2_HPO_4_, 1 g/L KH_2_PO_4_, 0.2 g/L MgSO_4_·7H_2_O, 0.05 g/L FeSO_4_, 0.035 g/L CaCl_2_. For solid agar plates, 20 g/L agar was added. As the strains were sediments when they were centrifuged and transferred to MSM, it was necessary to provide certain energy substances to maintain its initial growth [39,40]. Only a small amount of yeast extract (0.02 g/L) was added to the MSM to facilitate the growth of the strains. It was reported that adding a small amount of yeast extract to the MSM had a positive effect on the aromatic ring dioxygenase expressing bacteria, and then it can promote the strains to degrade targeted pollutants [41]. Before incubation, the pH was adjusted to 7.0–7.2, and the medium was autoclaved at 121 °C for 20 min.

### 2.2. Isolation and Identification of a Phenanthrene-Degrading Microbial Consortium

A PHE-degrading microbial consortium was obtained from PHE-contaminated soil (Da gang Oilfield, Tianjin, China). Soil samples were added to a 250 mL conical flask with 100 mL MSM which contained 100 mg/L PHE and cultured at 30 °C with shaking at 220 rpm for a week. Firstly, PHE was dissolved in n-hexane as the mother liquor, and then added into the MSM [42]. Since the mother liquor and MSM were immiscible with each other, they were clearly layered. After n-hexane volatilized completely, it was clearly observed that the layers disappeared, and white crystals formed on the surface of the MSM [29,43]. Then, 3% of the PHE enrichment culture was added to 100 mL MSM in a 250 mL conical flask and cultured at 30 °C with shaking at 220 rpm for a week. This enrichment step was repeated for 3 months. Then, the dilution plating method was used to isolate the single-strains. The enrichment culture was diluted to 10^−1^, 10^−2^, 10^−3^, 10^−4^, and 10^−5^, and spread on BPM, YPD, and LB agar plates. This is a planned method to obtain high biomass for subsequent identification and testing. Three different rich-nutrient media were used to cover the nutritional needs of microorganisms as much as possible. Finally, the strains were individually cultured in an incubator at 30 °C for 24 h.

Microscopic observation and 16S rDNA gene sequencing were performed on the strains that were screened. The bacterial genomic DNA extraction kit purchased from Tiangen Biochemical Technology Company (Beijing, China) was used to extract the genomic DNA of screened strains. The 16S rDNA genes were amplified using PCR with the universal bacterial primers 27F and 1492R. The amplification products were sequenced using GENEWIZ, Inc. (Beijing, China). The sequencing results were compared and analyzed using the BLAST program in the NCBI database (http://www.ncbi.nlm.nih.gov/blast/Blast.cgi) to determine the strain genus (accessed on 20 November 2021).

### 2.3. Structural Comparison of Microbial Consortia HJ-SH and HJ-7

The consortium, named HJ-7, consisted of seven strains in the same proportion. The consortia HJ-SH and HJ-7 were inoculated at 3% (*v*/*v*) into 100 mL of PHE degradation medium (100 mg/L), and incubated at 30 °C with shaking at 220 rpm for 3 days. The microorganisms in the reaction solution were transferred onto the surface of the filter paper, and the filter paper was sent to LC-Bio (Hangzhou, China) for microbial community diversity analysis.

### 2.4. Phenanthrene Biodegradation by Single Strains and Consortia

In this study, seven PHE-degrading strains were screened. The identified strains, SH-1, SH-2 and SH-3 were cultured in 5 mL BPM; SH-4 was cultured in 5 mL YPD medium; SH-5, SH-6 and SH-7 were cultured in 5 mL LB medium. These strains were cultured overnight, transferred to 100 mL BPM, YPD, and LB medium, respectively. Then, they were cultured at 30 °C with shaking at 220 rpm. When the OD_600_ was 0.8, the culture medium was centrifuged at 4 °C and 10,000× *g* for 10 min, and the supernatant was discarded. The sediment was washed three times and resuspend with sterile MSM. The suspension was added to the degradation medium of PHE as seed solution [39].

Using 100 mg/L PHE as the sole carbon source, the PHE degradation experiments of the screened single-strains and consortia were conducted. Specifically, we conducted PHE degradation experiments for single-strain, six-strains, the consortia HJ-SH and HJ-7 in order to determine which strain or the consortium played an effective role in PHE degradation. The specific combination of strains is shown in Table 1. Each group was inoculated at 3% (*v*/*v*) into 100 mL PHE degradation medium (100 mg/L), and incubated at 30 °C with shaking at 220 rpm for 3 days. In order to test the tolerance and degradation efficiency of HJ-SH for higher PHE concentrations, PHE was added to the MSM at 1, 2, 3, 4 and 4.5 g/L. Each group was inoculated into three conical flasks, and PHE degradation medium with sterile water inoculation was used as the control.

### 2.5. Biodegradation of Biphenyl, Naphthalene and n-Hexadecane

Briefly, 100 mg/L biphenyl, 100 mg/L naphthalene, and 100 mg/L n-hexadecane were added in 250 mL conical flasks containing 100 mL MSM, respectively. The cultures were inoculated with HJ-SH at 3% (*v*/*v*) and incubated at 30 °C with shaking at 220 rpm for 5 days to determine the degradation ratios. MSM-containing substrate with sterile water inoculation was used as the control.

### 2.6. Analysis of the Degradation Ratio

In order to determine the degradation ratio, the residual substrate PHE was extracted from the flask with an equal volume of n-hexane and filtered through a 0.22 µm organic membrane filter. Before extraction, 0.8 mg/mL pyrene was added to the sample as internal standard [44]. After extraction, the residual substrate was quantified using a gas chromatograph (GC-430, Bruker, Germany) equipped with a flame ionization detector (FID) and an RXI-1HT column (length 30 m; inner diameter 0.32 mm; film thickness, 0.25 µm). The carrier gas was nitrogen at a flow ratio of 1 mL/min. The injection volume was 1 µL and the split ratio was 1:1. The injector temperature was 280 °C. The column temperature was maintained at 80 °C for 5 min, then increased to 100 °C at a rate of 5 °C/min, and to 280 °C at a rate of 15 °C/min.

The extraction of PHE and sample pretreatment were conducted according to a published method [45]. The peak area, integral of the peak, was used to represent the total content of substrates. Each sample included a control group and an experimental group. Pyrene was used as the internal standard in the calculation of the residual peak area of each substrate to reduce error. The degradation ratio of each substrate was calculated by integrating the area of the internal standard peak and the area of the residual substrate. The degradation ratios of each substrate were calculated using the following formula:

$$Degradation\;ratio=\frac{AH\;control-\frac{AH\;experimental\times AT\;control}{AT\;experimental}}{AH\;control}$$

where AH is the peak area of the residual substrate, and AT is the peak area of the internal standard.

### 2.7. Statistical Analysis

All experiments were performed in triplicate, and the data presented are the averages of three values.

Statistical tests were carried out using the R software package (R foundation for statistical computing, version 4.0.5, Vienna, Austria). All tests were performed using nonparametric analysis (Kruskal–Wallis) with *p*-adjustment (Bonferroni) due to the small sample size (*n* = 3) and the heteroscedasticity of the data. Differences were regarded as statistically significant for all tests if *p* < 0.05.

## 3. Results

### 3.1. Isolation and Characterization of a Phenanthrene-Degrading Microbial Consortium

Using PHE as the carbon source, the natural PHE-degrading microbial consortium HJ-SH was obtained from PHE-contaminated soil samples through multiple rounds of enrichment culture. Seven strains were isolated from HJ-SH, and named as SH-1 to SH-7, respectively. SH-1, SH-2, SH-3 and SH-5 were Gram-negative bacteria, while SH-4, SH-6 and SH-7 were Gram-positive bacteria. SH-1 was yellow, transparent, and regular round in shape; SH-2 and SH-6 were white, opaque, glossy, and regular round in shape; SH-3 was brown, opaque, glossy, and regular round in shape; SH-4 was yellow, opaque, glossy, and regular round in shape; SH-5 was white, opaque, and regular round with obvious boundary; SH-7 was brownish yellow, opaque, shiny, and irregular in shape. The seven strains were identified as *Pseudomonas* sp., *Stenotrophomonas* sp., *Delftia* sp., *Pseudomonas* sp., *Brevundimonas* sp., *Curtobacterium* sp., and *Microbacterium* sp., respectively. The results of Gram-staining and 16S rDNA gene sequences of the seven strains are shown in Supplementary Information.

### 3.2. Structural Comparison of Microbial Consortia HJ-SH and HJ-7

**Figure 1:**
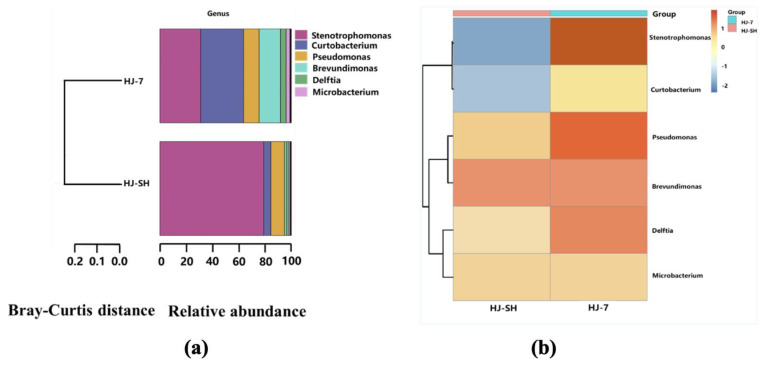
(**a**) Diagram of relative genus abundance of consortia HJ-SH and HJ-7. The width of the block represents the relative genus abundance. The Bray–Curtis distance on the left represents similarity of genus composition in different groups. (**b**) The clustering heat map analyzed using the 16S rDNA. The clustering branches on the left represent cluster results. The values corresponding to different colors of the middle square correspond to the values Z of the relative genus abundance in each row after standardization which makes horizontal comparison meaningful whereas vertical comparison meaningless.

We analyzed the 16S rDNA gene sequencing results of consortia HJ-7 and HJ-SH after degrading PHE for 3 days. As shown in Figure 1a, the relative genus abundance of consortia HJ-SH and HJ-7 was different. The initial proportions of the seven single strains in consortium HJ-7 were the same. The proportion of genera *Stenotrophomonas* and *Curtobacterium* significantly increased, while the other four genera significantly decreased after degrading PHE for 3 days. The relative genus abundance analysis results of consortium HJ-SH indicated that the proportion of genus *Stenotrophomonas* was the highest; it was extremely higher than other genera. As shown in the clustering heat map of Figure 1b, the difference between the relative abundance A_1i_ of a sample in a certain genus classification and the average A_i_ of all samples in a certain genus classification divided by the standard deviation σ_i_ of all samples in a certain genus classification was value Z. Comparing Figure 1a,b, changes in the relative genus proportion were found. Taking the genus *Stenotrophomonas* as an example, if all the strains in consortium HJ-SH are isolated and there are no strains except these seven strains, the Z value should be positive. However, the color of the square was blue, indicating a negative Z value, which indicated that there may be large amounts of *Stenotrophomonas* strains in consortium HJ-SH that cannot be purely cultured. The evidence in other recent and reliable studies supported the conclusion that most bacterial and archaeal taxa remained uncultured [46,47], largely owing to challenges in cultivating microorganisms under laboratory conditions [48].

### 3.3. Characterization of Phenanthrene Biodegradation

#### 3.3.1. Phenanthrene Degradation by HJ-7 and HJ-SH

**Figure 2:**
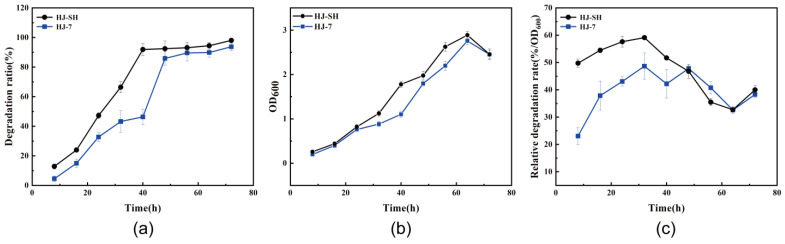
(**a**) The PHE degradation ratio of the consortia HJ-7 and HJ-SH within 72 h. (**b**) Growth curve of the consortia HJ-7 and HJ-SH within 72 h. (**c**) The relative PHE degradation rate (ratio of degradation ratio to OD_600_) of the consortia HJ-7 and HJ-SH within 72 h. Data are expressed as means ± standard deviations of three independent replicates.

The PHE degradation ratios and strain growth of consortia HJ-SH (Figure S1) and HJ-7 with 3% (*v*/*v*) inoculation were measured every 8 h for a total of 3 days. As shown in Figure 2a, the degradation ratio of the consortium HJ-SH reached 13% at 8 h and rose to 92% at 40 h. Finally, the degradation ratio of the consortium HJ-SH reached 98% at 72 h. The degradation ratio of the consortium HJ-7 increased slowly in the first 40 h, reaching 46% at 40 h. The fastest growth period of degradation ratio is 40–48 h, during which the degradation ratio increased from 46% to 86%. Finally, the degradation ratio of the consortium HJ-7 reached 94% at 72 h, and the final degradation ratios of the two consortia were similar under the same conditions. As shown in Figure 2b, OD_600_ was chosen to represent the microbial growth state. The results of this method are accurate within a limited range (0.2–0.8), and accordingly, it needs a precise and laborious calibration. It can be noticed that the colored and suspended intermediate products produced during the degradation of PHE that cannot be separated from cells may cause an increase in OD_600_. But using colony counting to determine the number of living cells also has problems such as long detection cycle and large human operation error [49]. On the other hand, the consortia consist of seven or more strains, making the direct counting challenging. All things considered, OD_600_ is appropriate to measure the growth state in this study. The growth curve showed that the consortia HJ-SH and HJ-7 both grew relatively slowly in the first 24 h and began to grow rapidly after 24 h. One or several dominating degrading strains in consortium HJ-7 grew rapidly within 40–48 h, resulting in a sharp increase in degradation ratio during this period. The two consortia had continued to grow rapidly but the degradation ratios had increased slowly within 48–60 h, which indicated that the dominant strains during this period had little impact on PHE degradation, and they were using the intermediate products of PHE degradation process for growth. Both consortia entered the decline phase after 64 h. The growth curve was basically consistent with the phenomena observed in the degradation process. In addition, we calculated the relative degradation rates of the two consortia, which represented the specific degradation rate per unit of OD_600_. As shown in Figure 2c, the overall trends of relative degradation rates of the two consortia were to increase first and then decrease. Due to the same initial proportion of strains in HJ-7, some of the seven degrading bacteria did not dominate before a certain period of community succession. In contrast, HJ-SH had completed community succession during the domestication process. Therefore, the relative degradation rate of HJ-SH was significantly higher than that of HJ-7 before 48 h. After 48 h, the relative degradation rate of HJ-7 and that of HJ-SH were almost identical, which indicated that the proportion of strains in the consortium HJ-7 was appropriate after community succession. In the process of PHE degradation, there may be some differences in the degradation ratios, OD_600_ and relative degradation rate of consortia HJ-SH and HJ-7, due to the differences in the composition and succession of microbial communities. But the trend of the three parameters in the two microbial consortia were extremely similar, especially in the degradation telophase. All results indicated that the two consortia are very similar, which further verified that the seven strains were the dominating degrading strains in the consortium HJ-SH.

#### 3.3.2. Phenanthrene Degradation by Single-Strain and HJ-SH

**Figure 3:**
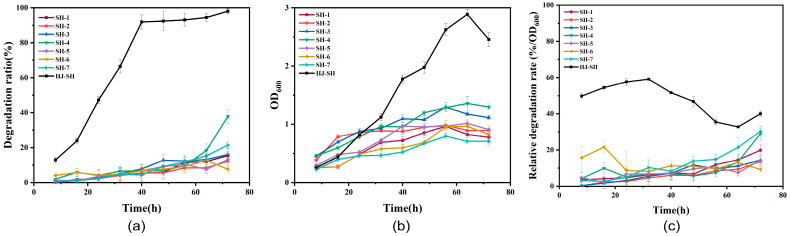
(**a**) The PHE degradation ratio of the strains SH-1, SH-2, SH-3, SH-4, SH-5, SH-6, SH-7 and the consortium HJ-SH within 72 h. (**b**) Growth curve of the single-strains and the consortium HJ-SH within 72 h. (**c**) The relative degradation rate of the single-strains and the consortium HJ-SH within 72 h. Data are expressed as means ± standard deviations of three independent replicates.

To find out the role played by each single-strain in the PHE degradation process, the degradation ratios and growth of single-strains and consortium HJ-SH were measured every 8 h for a total of 3 days. As shown in Figure 3a, the degradation ratios of the seven strains were 15%, 12%, 16%, 38%, 13%, 8% and 21%, respectively. The degradation ratios of single-strains were all extremely lower than that of HJ-SH. By contrast, the PHE-degrading strain *Zhihengliuella* sp. ISTPL4, isolated from sediment samples of a lake in India, removed 81 and 87% of 100 mg/L PHE after 72 and 168 h, respectively [50]. An alkaliphilic, metallotolerant bacterium, *Pseudomonas aeruginosa* san ai, degraded PAHs (PHE, pyrene) with ratios of 50% and 41%, respectively, under initial concentrations of 20 mg/L and in 7 days [33]. It can be determined that the individual degradation ability of single-strains is not outstanding, but the degradation ability of consortium HJ-SH is excellent. As shown in Figure 3b, the OD_600_ of HJ-SH was higher than that of single-strains, although the inoculation amounts were the same. In the first 40 h, the consortium HJ-SH was in the logarithmic growth phase, so the degradation was mainly used for microbial growth. A significant positive relationship was found between microbial growth and PHE degradation in prior research [51]. It has also been demonstrated in this study that the degradation ratio and OD_600_ of SH-4 are both the highest among the single-strains. In addition, significant differences of relative degradation rates between single-strains and consortium HJ-SH can be found in Figure 3c. The relative degradation rate of consortium HJ-SH was much higher than those of single-strains, which indicated that the degradation efficiency presented by the same biomass in the consortium HJ-SH with more cooperative actions was significantly better than single-strains. Specially, there was no significant difference in the relative degradation rates between SH-4 and single-strains, so it was speculated that by increasing the biomass rather than improving the degradation efficiency, degradation contribution of SH4 could be enhanced.

#### 3.3.3. Phenanthrene Degradation by Six-Strains and HJ-SH

**Figure 4:**
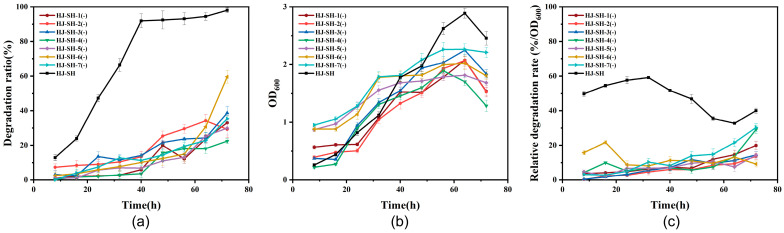
(**a**) The PHE degradation ratio of the consortia HJ-SH-1(-), HJ-SH-2(-), HJ-SH-3(-), HJ-SH-4(-), HJ-SH-5(-), HJ-SH-6(-), HJ-SH-7(-) and HJ-SH within 72 h. (**b**) Growth curve of the six-strain consortia and the consortium HJ-SH within 72 h. (**c**) The relative degradation rate of the six-strain consortia and the consortium HJ-SH within 72 h. Data are expressed as means ± standard deviations of three independent replicates.

Since the degradation efficiency of the consortium HJ-SH was much better than that of single-strains, we decided to further determine which combination of strains was optimal. The optimal combination should have the characteristics of small size, high efficiency and stability. First, we removed one strain from the consortium HJ-7 to compare the changes in PHE degradation ratio. As shown in Figure 4a, the PHE degradation ratios of HJ-SH-1(-), HJ-SH-2(-), HJ-SH-3(-), HJ-SH-4(-), HJ-SH-5(-), HJ-SH-6(-), and HJ-SH-7(-) were 33%, 30%, 39%, 22%, 26%, 60% and 35%, respectively. The degradation ratios of each six-strain consortium seemed to be higher than that of single-strains, but much lower than that of the consortium HJ-SH. However, these differences were not simple instances of addition or subtraction. Regardless of which single-strain was removed, the degradation ratio was significantly reduced. According to the report, degradation was usually carried out by metabolic cooperation within a microbial consortium rather than by one degrading strain [52]. The cooperation and symbiosis of the strains in a consortium enhance their degradation ability [53]. According to the experimental comparison, the consortium HJ-SH was the best PHE-degrading microbial consortium obtained in this study. As presented in Figure 4b, the growth trends of the six-strains were basically consistent with the trends of degradation ratio. Notably, both the degradation ratio and OD_600_ of the consortium HJ-SH-4(-) seemed to be the least among the six-strain consortia, which indicated SH-4 was very important. Similarly, significant differences in the relative degradation rates between six-strains and consortium HJ-SH can be also found in Figure 4c. The relative degradation rates of six-strains were much lower than that of consortium HJ-SH, and were also much lower than that of consortium HJ-7 (compared with Figure 2c), which indicated that the absence of any of the seven dominating strains resulted in a significant decrease in degradation efficiency.

#### 3.3.4. The Contribution of the Seven Strains

**Figure 5:**
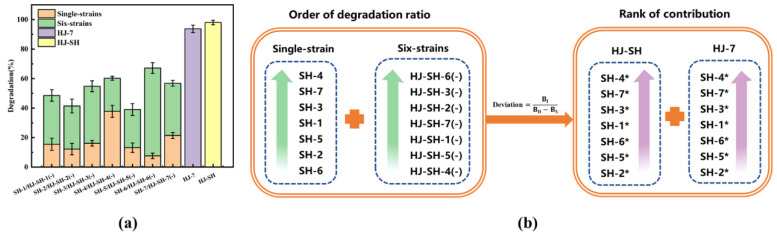
(**a**) Comparison of PHE degradation ability among the single-strains and the six-strain consortia, the consortia HJ-7 and HJ-SH under the same condition. (**b**) Order of degradation ratio and rank of contribution. According to the contribution formula, contribution of the seven strain in PHE degradation is ranked. B_H_: degradation ratio of the consortium HJ-SH or HJ-7; B_S_: degradation ratio of the six-strain consortia; B_I_: degradation ratio of the single strains. Data are expressed as means ± standard deviations of three independent replicates.

The consortium HJ-SH was obtained from the PHE-contaminated soil, and then, seven single strains, SH-1 to SH-7, were isolated from the consortium. In order to find out the degradation ability of each strain and whether or not the seven strains in the consortium degrade PHE synergistically, PHE-degrading experiments were conducted for the single-strain and six-strain consortia, the consortia HJ-7, and HJ-SH; other aims of the tests were to find out if each of the seven strains was indispensable and what was the contribution of each strain to the whole consortium. To compare the importance of each strain, the contribution of one single-strain to the consortium HJ-SH or HJ-7 was inferred by the ratio of the actual degradation ratio (BI) to the potential degradation ratio (BH-BS), where BI, BH, and BS presented the degradation ratio of the single-strain, the six-strain consortia, the consortia HJ-7 or HJ-SH, respectively. The values of contribution of each strain to the consortium HJ-SH or HJ-7 were listed in Table 2. The closer the value is to 1, the more fully the degradation ability of single-strain is shown, and the more important the single-strain is. As shown in Figure 5a, according to the order of degradation ratio of single-strain and six-strains, the degradation ratio of the consortia HJ-SH and HJ-7 were much better than those of other combinations, which proved that these seven strains cooperated to degrade PHE and each strain was indispensable. In Table 2 and Figure 5b, SH-4 showed the highest value of contribution and SH-2 showed the minimum, and the results in the consortia HJ-SH and HJ-7 were the same, which further proved the similarity between the consortia HJ-SH and HJ-7. The single-strains SH-4 and SH-6 were two extremes. The single-strain SH-4 showed the strongest degradation ability and the degradation ability of the consortium HJ-SH-4(-) significantly weakened after the strain SH-4 was removed from it. This result confirmed the previous speculation that SH-4 was the most important strain. Additionally, the single-strain SH-6 showed the weakest degradation ability, and SH-2 had the smallest degradation contribution, while the other five strains showed no clear order of degradation ability or degradation contribution. In previous studies, *Pseudomonas* strains showed significant production of surface-active compounds, and a strong chemotactic response toward PHE [54]. *Pseudomonas* sp. were able to degrade PAHs and convert them into medium-chain-length(mcl)-PHA [55]. *Pseudomonas* sp. USTB-RU showed the potential to produce surface-active compounds that may improve biodegradation by enhancing the substrate bioavailability [56]. These results explained why SH-4 had the strongest degradation ability. However, the relationship between each strain in the consortium HJ-SH is very complex, and further research should be carried out later. Based on the above analysis, we can conclude that low degradation ratio or contribution degree of a certain strain do not mean that it is not “important”. The synergy between these seven strains is the main factor leading to the very efficient PHE degradation by the consortium HJ-SH.

#### 3.3.5. PHE Tolerance of the Consortium HJ-SH

**Figure 6:**
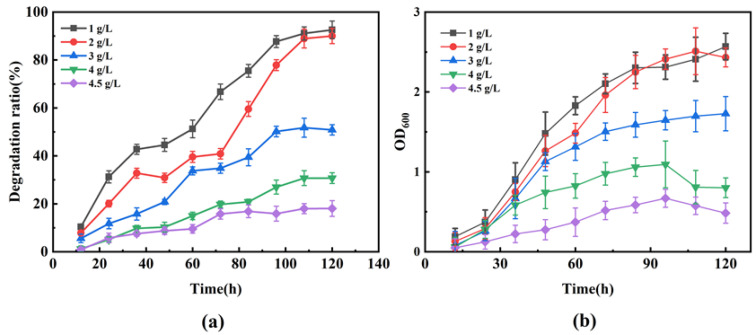
(**a**) The PHE degradation ratio of the consortium HJ-SH under PHE concentrations of 1, 2, 3, 4, and 4.5 g/L within 5 days. (**b**) Growth curve of the consortium HJ-SH under PHE concentrations of 1, 2, 3, 4, and 4.5 g/L within 5 days. Data are expressed as means ± standard deviations of three independent replicates.

Since HJ-SH exhibited an excellent ability to degrade 100 mg/L PHE, we further tested its tolerance to higher PHE concentrations. When the PHE concentration was 5 g/L, it was observed that PHE on the upper layer of the degradation medium was not degraded, and the strains did not grow in the medium in 5 days. Preliminary experiment phenomenon indicated that PHE could not be degraded when its concentration was above 5 g/L. In general, the solubility of the organic matter is very small. In most cases (concentration of the organic matter far beyond its saturation solubility), the degradation system exists in the form of an oil–water phase, and the degradation of microorganisms occurs at the oil–water phase interface. PHE is the organic phase and exists in the form of crystal in the MSM as observed. When the concentration of organic matter increases, the crystal becomes larger, the specific surface area becomes less, the available organic matter becomes less, and the inhibition of microorganisms is greater. At a certain concentration, microorganisms are completely inhibited [57]. The PHE concentrations, 1, 2, 3, 4, and 4.5 g/L, were chosen to carry out the tolerance experiment. As shown in Figure 6a, in the medium containing 1, 2, 3, 4, and 4.5 g/L PHE, the degradation ratios of the microbial consortium HJ-SH ranged from 18 to 93% in 5 days. The final degradation ratio decreased with the increase in the initial substrate concentration. The maximal PHE tolerance of the consortium HJ-SH was 4.5 g/L. In Figure 6b, when the initial concentrations ranged from 1 to 4.5 g/L, the final OD_600_ decreased with the increase in the initial substrate concentration, while the growth trends of microorganisms with time course became flatter under higher initial substrate concentration. The substrate was restrictive under lower concentrations and inhibitory under higher concentrations, resulting in the differences of final degradation ratio and OD_600_. As seen, the PHE degradation ratio was positively correlated with cell growth. By contrast, the PHE tolerance of *Trichoderma* was 3 g/L [57] and that of *Acidovorax* sp. JG5 was 1.5 g/L [29]. Notably, the 1 g/L PHE degradation ratio of the consortium HJ-SH was the highest, which reached 93% in 5 days. And the consortium HJ-SH showed excellent PHE degradation ability and tolerance, which may be due to the adaptation and symbiosis of the seven strains enhancing the tolerance to organic pollutants [45].

### 3.4. Degradation of Other Organic Pollutants

**Figure 7:**
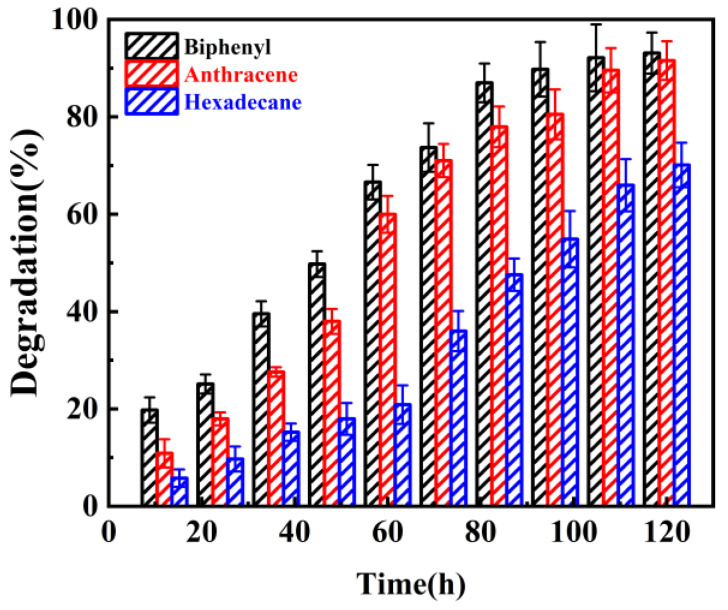
Other organic pollutants degradation ratio of the consortium HJ-SH within 5 days. The degradation efficiency was in the following order: biphenyl > anthracene > n-hexadecane. Data are expressed as means ± standard deviations of three independent replicates.

Finally, we studied the ability of microbial consortium HJ-SH to degrade other organic pollutants. Anthracene and biphenyl belong to PAHs, and n-hexadecane belongs to long-chain alkanes (a typical petroleum pollutant). The main reason we study these substances is because their degradation pathways are partly the same as the metabolic pathway (or typical, such as n-hexadecane). Secondly, these substances are included in common petroleum pollutants. Results showed that the consortium HJ-SH could degrade biphenyl, anthracene, and n-hexadecane. As can be seen in Figure 7, the degradation ratios of 100 mg/L biphenyl, anthracene and n-hexadecane by HJ-SH reached 93%, 92% and 70%, respectively, after 5 days. Commonly, the degradation ratio of chain alkanes is higher than that of aromatic hydrocarbons by the same strains. But there are some exceptions; the degradation ratio of the former is lower than that of the latter. In our research, the consortium HJ-SH had the best ability to degrade biphenyl and the worst ability to degrade n-hexadecane. And it may be that the synergistic effect of the seven strains can degrade bicyclic and tricyclic PAHs; nevertheless, their ability to degrade n-hexadecane is relatively lower. A similar phenomenon has been reported, wherein a newly isolated bacterium had the ability to degrade PAHs but cannot use n-hexadecane and octadecane as the carbon and energy source [58]. The surface hydrophobicity of five PAH-degrading yeasts was 91.6–97.5% when the substrate was naphthalene, but 82.0–97.4% when the substrate was n-hexadecane [59]. The results show that the consortium HJ-SH can degrade these organics, indicating that HJ-SH has great potential for the degradation of pollutants and can be better applied in field bioremediation.

## 4. Discussion

PHE is widely researched as one of the model compounds for the study of biodegradation of PAHs. In the present study, the microbial consortium HJ-SH, isolated from long-term PHE-contaminated soil, showed a robust range of growth conditions, excellent tolerance to PHE, and extremely high PHE degradation ratios. At present, the studies on the degradation of PHE are mostly based on PHE-degrading mechanisms of single-strains and the addition of ancillary substrates or surfactants to improve the degradation. There are also some studies on natural and artificial consortia, but the number of consortium members is limited, usually two to three, due to the complexity of the structure and function of consortia which contain more members. As shown in Table 3, *Sphingobium xenophagum* D43FB showed a great PHE degradation ability, degrading 95% of the initial 50 mg/L PHE [60]. After 21 days, *Cladosporium* sp. CBMAI 1237 degraded only 47% of 100 mg/L PHE [61]. To our knowledge, the engineered strain, *Pseudomonas* sp. CGMCC2953-pK, showed the highest degradation ratio and the best degradation ability, which degraded 98% of 100 mg/L PHE after 7 days. According to the PHE degradation efficiency, the natural consortium HJ-SH and the artificial consortium HJ-7 are the best two microbial consortia obtained in this study. The consortium HJ-SH degraded 98% of 100 mg/L PHE within 72 h, and the consortium HJ-7 degraded 94%. In contrast to other single-strains and consortia reported, these two consortia showed significant PHE degradation ability, and their efficiency was higher than that of any other natural consortia reported so far, even higher than most of the engineered strains and consortia. Additionally, HJ-SH showed much greater tolerance to PHE.

According to the study on the degradation of PHE by different combinations, it was concluded that SH-4 showed the strongest degradation ability, and SH-6 showed the weakest degradation ability; SH-4 had the biggest degradation contribution, and SH-2 had the smallest degradation contribution. It is suspected that there are key genes in SH-4 that play a crucial role in the PHE degradation process. In previous research, the complete metabolic pathways of PHE were reported: ring cleavage pathway, salicylic acid pathway or phthalic acid pathway, and TCA cycle [62,63]. Dioxygenase, salicylate hydroxylase and catechol 1,2-dioxygenase are the crucial enzymes for degrading PHE [36]. It is speculated that under the synergistic effect of other strains in the consortia, the following factors make HJ-4 unusual: better expression of key genes, higher activity of key enzymes, higher metabolic flux and potential for producing surfactants. However, compositions of the two consortia obtained in this study are very complex, meaning more unpredictable succession of microbial communities. Therefore, it is very challenging to analyze the metabolic mechanism of a single-strain in the presence of multiple strains, which is one of our future research directions.

The natural consortium HJ-SH and the artificial consortium HJ-7 are the best two PHE-degrading microbial consortia in this study, especially HJ-SH which shows the best degradation ability and the highest tolerance of PHE. Although the laws of natural microbial community succession can be analyzed, the succession is difficult to be controlled artificially [64]. For example, the genus *Stenotrophomonas* of the consortium HJ-SH is at a position of advantage in Figure 1a, which indicates that long-term storage may lead to unstable degradation ability. The artificial consortium HJ-7 is relatively easy to replicate compared to the natural consortium HJ-SH and has stronger application stability. The results of degradation experiment with different combinations indicate that consortium HJ-7 contains seven crucial strains, in which these is no redundant strain. Moreover, the degradation ratios, growth trends and the relative degradation rates of the two microbial consortia are extremely similar. As a microbial consortium without engineering, HJ-7 has significant PHE degradation ability and a strong application stability. Therefore, we believe that the artificial consortium HJ-7 has more field application potential, and it can replace the consortium HJ-SH to a great extent. Further genetic engineering modifications are under consideration.

**Table 3:** Studies on PHE degradation.

Strain Name	The Ability to Degrade PHE	Reference
*Coriolopsis byrsina*	Degraded 99.90% of 20 mg/L PHE under in vitro conditions and 77.48% of 50 mg/L PHE under in vivo conditions.	[35]
*Kocuria flava,* *Rhodococcus pyridinivorans*	*K. flava* could degrade PHE with an efficiency of 55.13%, *R. pyridinivorans* exhibited 62.03% efficiency when the PHE concentration was 10 mg/L.	[65]
*Serratia marcescens*	Degraded 44% of 100 mg/L PHE after 3 days.	[66]
*Pseudomonas* sp. VB92 *Bacillus* sp. JK17	Degraded 54.21% of 200 mg/L PHE in 10 days and 59.91% in 12 days.	[67]
*Sphingobium xenophagum D43FB*	Degraded 95% of 50 mg/L PHE after 5 days.	[60]
*Cladosporium* sp. CBMAI 1237	Degraded 47% of 100 mg/L PHE and other PAHs after 21 days.	[61]
*Pseudomonas* sp. CGMCC2953-pK (engineered strain)	Degraded 98% of 100 mg/L PHE after 7 days.	[68]
Consortium YL (wild consortium)	Degraded 92.3% of 50 mg/L PHE after 21 days.	[69]
Halothermophilic consortium (engineered consortium)	Degraded 58% of 200 mg/L PHE after 42 days.	[70]
Artificial *E. coli* BL21 consortium (engineered consortium)	Degraded 90.66% of 100 mg/L PHE after 21 days.	[36]
Consortium HJ-SH (wild consortium)	Degraded 98.06% of 100 mg/L PHE after 3 days.	Present study
Consortium HJ-7 (artificial consortium without engineering)	Degraded 93.75% of 100 mg/L PHE after 3 days.	Present study

## 5. Conclusions

In conclusion, we isolated a natural PHE-degrading microbial consortium HJ-SH with very high degradation efficiency from PHE-contaminated soil via multiple rounds of domestication and screening. The consortium HJ-SH degraded 98% of 100 mg/L PHE in 3 days and 93% of 1000 mg/L PHE in 5 days, an efficiency which is higher than that of any other natural consortia reported so far, even higher than most of the engineered strains and consortia. In addition, it can tolerate up to 4.5 g of PHE, and effectively degrade biphenyl, anthracene, and n-hexadecane. Moreover, we cultured seven pure strains from the natural consortium HJ-SH, and accordingly constructed an artificial consortium HJ-7 with these seven strains. By comparing the degradation ratios, growth and relative degradation rates of different combinations and single strains, the following conclusions were drawn: (1) There still might be unidentified microorganisms in the consortium HJ-SH, but the seven purely cultured strains are dominating degrading strains, among which SH-4 is the most important in PHE degradation. (2) Consortium HJ-7 can largely replace consortium HJ-SH due to their similarity in PHE degradation ability, easier reproducibility, better application stability, and larger room for modification. The high degradation potential and broad application prospects of natural microorganisms from polluted sites as well as artificial consortia based on these microorganisms have been proven by this research, which now provide novel tools and new insights for the bioremediation of PAHs and other organic pollutants by microbial consortia.

## Figures and Tables

**Table 1:** Four different combinations of strains.

Strain Combination	Source	Name
Microbial consortium	PHE-contaminated soil	HJ-SH
Single-strain pure cultures	Seven single-strains isolated from HJ-SH	SH-1, SH-2, SH-3, SH-4, SH-5, SH-6, SH-7
Six-strain co-cultures	Remove SH-1 from HJ-SH	HJ-SH-1 (-)
	Remove SH-2 from HJ-SH	HJ-SH-2 (-)
	Remove SH-3 from HJ-SH	HJ-SH-3 (-)
	Remove SH-4 from HJ-SH	HJ-SH-4(-)
	Remove SH-5 from HJ-SH	HJ-SH-5(-)
	Remove SH-6 from HJ-SH	HJ-SH-6(-)
	Remove SH-7 from HJ-SH	HJ-SH-7(-)
Seven-strain co-culture	Seven strains isolated from HJ-SH	HJ-7

**Table 2:** Results of the contribution of seven dominating strains in the consortium HJ-7 and HJ-SH.

Strains	SH-4	SH-7	SH-3	SH-1	SH-6	SH-5	SH-2
Contribution of HJ-SH	0.500	0.342	0.271	0.238	0.197	0.181	0.177
Contribution of HJ-7	0.530	0.367	0.290	0.255	0.222	0.193	0.189

## Data Availability

The data presented in this study are available in Supplementary Materials.

## Supplementary Materials

The following supporting information can be downloaded at: https://www.mdpi.com/article/10.3390/microorganisms11102383/s1, Figure S1. Changes in culture medium within 72 h; Figure S2. Plate underlined map of seven single-strains; Figure S3. Gram staining results of seven single-strains; 16S rDNA gene sequence; Table S1. Data of PHE degradation ratio; Table S2. Data of OD600; Table S3. Data of relative PHE degradation rate; Table S4. Data of PHE tolerance experiment; Table S5. Data of degradation ratio.
