# Supplementary material for: Microbial Consortium HJ-SH with Very High Degradation Efficiency of Phenanthrene
Source: Microorganisms. 2023 Sep 23;11(10):2383. doi: 10.3390/microorganisms11102383 (PMC10609217; doi:10.3390/microorganisms11102383)
Supplement: Supplementary file 1 [file microorganisms-11-02383-s001.zip › microorganisms-2575313-supplementary.pdf]

Supplementary Material

S1.1. Supplementary Figures

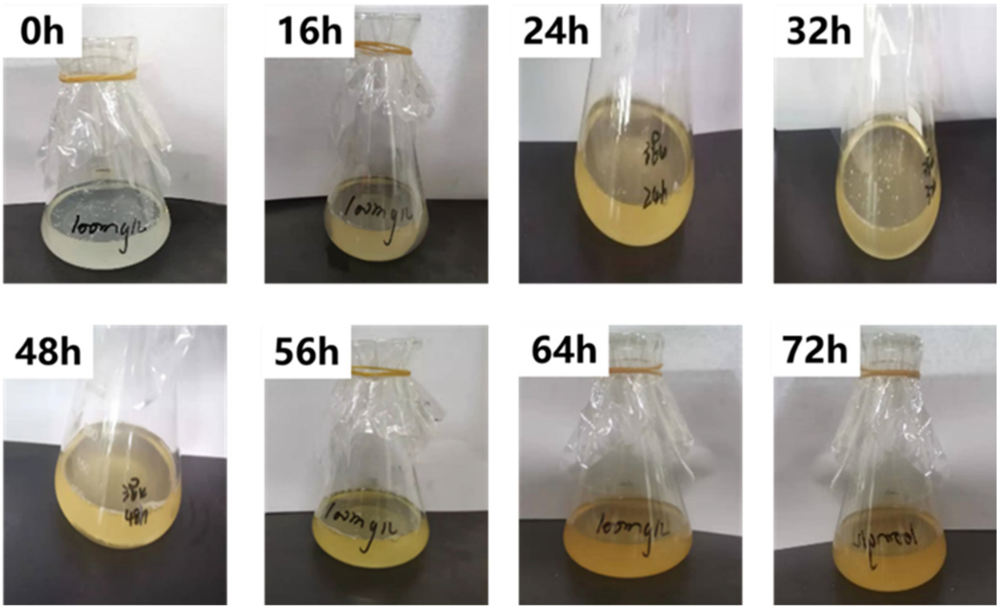

Supplementary Figure S1. Changes in culture medium within 72h.

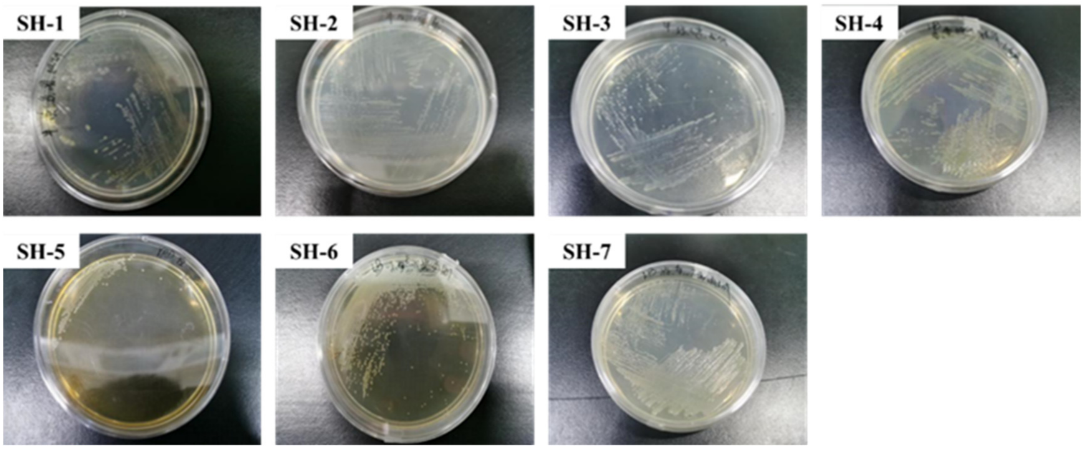

Supplementary Figure S2. Plate underlined map of seven single-strains.

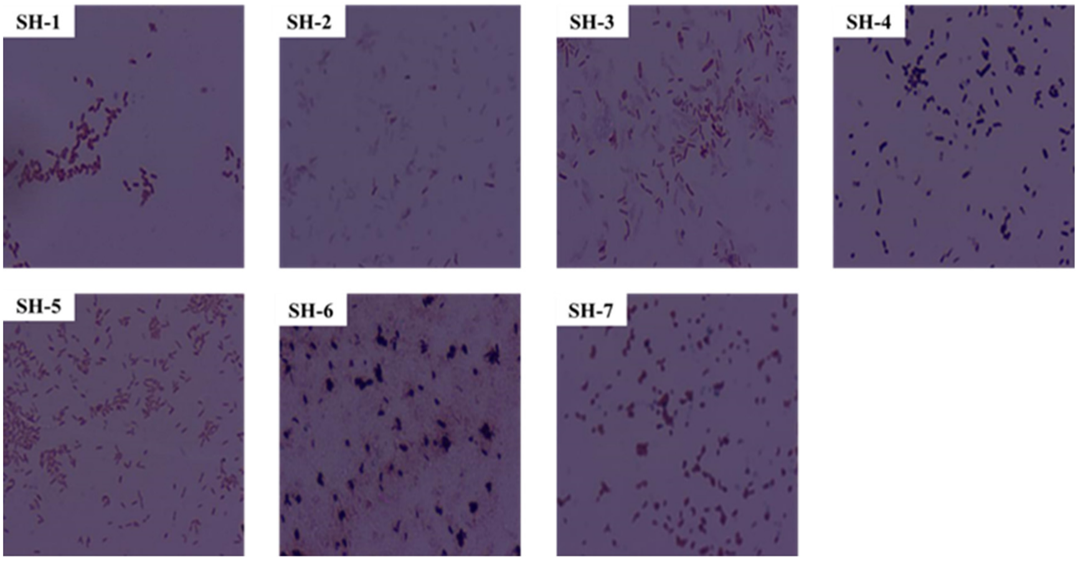

Supplementary Figure S3. Gram staining results of seven single-strains.

S1.2. Supplementary information

16S rDNA gene sequence

SH-1

Accession number: OR241333

TTTCCCCGTTGGTTACCGTCCTCCCGAAGGTTAGACTAGCTACTTCTGGTGCAACCCACT
CCCATGGTGTGACGGGCGGTGTGTACAAGGCCCGGGAACGTATTCACCGCGACATTCTG
ATTCGCGATTACTAGCGATTCCGACTTCACGCAGTCGAGTTGCAGACTGCGATCCGGACT
ACGATCGGTTTTGTGAGATTAGCTCCACCTCGCGGCTTGGCAACCCTCTGTACCGACCAT
TGTAGCACGTGTGTAGCCCAGGCCGTAAGGGCCATGATGACTTGACGTCATCCCCACCT
TCCTCCGGTTTGTCACCGGCAGTCTCCTTAGAGTGCCCACCATAACGTGCTGGTAACTAA
GGACAAGGGTTGCGCTCGTTACGGGACTTAACCCAACATCTCACGACACGAGCTGACGA
CAGCCATGCAGCACCTGTGTCAGAGTTCCCGAAGGCACCAATCCATCTCTGGAAAGTTC
TCTGCATGTCAAGGCCTGGTAAGGTTCTTCGCGTTGCTTCGAATTAAACCACATGCTCCA
CCGCTTGTGCGGGCCCCCGTCAATTCATTTGAGTTTTAACCTTGCGGCCGTACTCCCCAG
GCGGTCAACTTAATGCGTTAGCTGCGCCACTAAAATCTCAAGGATTCCAACGGCTAGTT
GACATCGTTTACGGCGTGGACTACCAGGGTATCTAATCCTGTTTGCTCCCCACGCTTTCG
CACCTCAGTGTCAGTATCAGTCCAGGTGGTCGCCTTCGCCACTGGTGTTCCTTCCTATAT
CTACGCATTTCACCGCTACACAGGAAATTCCACCACCCTCTACCGTACTCTAGCTTGCCA
GTTTTGGATGCAGTTCCCAGGTTGAGCCCGGGGCTTTCACATCCAACTTAACAAACCACC
TACGCGCGCTTTACGCCCAGTAATTCCGATTAACGCTTGCACCCTCTGTATTACCGCGGC
TGCTGGCACAGAGTTAGCCGGTGCTTATTCTGTCGGTAACGTCAAAACAGCAAGGTATT
AACTTACTGCCCTTCCTCCCAACTTAAAGTGCTTTACAATCCGAAGACCTTCTTCACACA
CGCGGCATGGCTGGATCAGGCTTTCGCCCATTGTCCAATATTCCCCACTGCTGCCTCCCG

TAGGAGTCTGGACCGTGTCTCAGTTCCAGTGTGACTGATCATCCTCTCAGACCAGTTACG
GATCGTCGCCTTGGTGAGCCATTACCCCACCAACTAGCTAATCCGACCTAGGCTCATCTG
ATAGCGCAAGGCCCGAAGGTCCCCTGCTTTCTCCCGTAGGACGTATGCGGTATTAGCGTT
CCTTTCGAAACGTTGTCCCCCACTACCAGGCAGATTCCTAGGCATTACTCACCCGTCCGC
CGCTGAATCAAGGAGCAAGCTCCCGTCATCCGCTCGACTTGCATGTGTTAGGCCTGCCG
CCAGCGTTCATCTGACGGGGGGTTTAT

SH-2

Accession number: OR241334

TGGGCAGGCTACCATGCAAGTCGAACGGCAGCACAGGAGAGCTTGCTCTCTGGGTGGCG
AGTGGCGGACGGGTGAGGAATACATCGGAATCTACTTTTTCGTGGGGGATAACGTAGGG
AAACTTACGCTAATACCGCATACGACCTACGGGTGAAAGCAGGGGATCTTCGGACCTTG
CGCGATTGAATGAGCCGATGTCGGATTAGCTAGTTGGCGGGGTAAAGGCCCACCAAGGC
GACGATCCGTAGCTGGTCTGAGAGGATGATCAGCCACACTGGAACTGAGACACGGTCCA
GACTCCTACGGGAGGCAGCAGTGGGGAATATTGGACAATGGGCGCAAGCCTGATCCAG
CCATACCGCGTGGGTGAAGAAGGCCTTCGGGTTGTAAAGCCCTTTTGTTGGGAAAGAAA
TCCAGCTGGTTAATACCCGGTTGGGATGACGGTACCCAAAGAATAAGCACCGGCTAACT
TCGTGCCAGCAGCCGCGGTAATACGAAGGGTGCAAGCGTTACTCGGAATTACTGGGCGT
AAAGCGTGCGTAGGTGGTCGTTTAAGTCCGTTGTGAAAGCCCTGGGCTCAACCTGGGAA
CTGCAGTGGATACTGGACGACTAGAGTGTGGTAGAGGGTAGCGGAATTCCTGGTGTAGC
AGTGAAATGCGTAGAGATCAGGAGGAACATCCATGGCGAAGGCAGCTACCTGGACCAA
CACTGACACTGAGGCACGAAAGCGTGGGGAGCAAACAGGATTAGATACCCTGGTAGTC
CACGCCCTAAACGATGCGAACTGGATGTTGGGTGCAATTTGGCACGCAGTATCGAAGCT
AACGCGTTAAGTTCGCCGCCTGGGGAGTACGGTCGCAAGACTGAAACTCAAAGGAATTG
ACGGGGGCCCGCACAAGCGGTGGAGTATGTGGTTTAATTCGATGCAACGCGAAGAACCT
TACCTGGCCTTGACATGTCGAGAACTTTCCAGAGATGGATTGGTGCCTTCGGGAACTCG
AACACAGGTGCTGCATGGCTGTCGTCAGCTCGTGTCGTGAGATGTTGGGTTAAGTCCCG
CAACGAGCGCAACCCTTGTCCTTAGTTGCCAGCACGTAATGGTGGGAACTCTAAGGAGA
CCGCCGGTGACAAACCGGAGGAAGGTGGGGATGACGTCAAGTCATCATGGCCCTTACG
GCCAGGGCTACACACGTACTACAATGGTAGGGACAGAGGGCTGCAAGCCGGCGACGGT
AAGCCAATCCCAGAAACCCTATCTCAGTCCGGATTGGAGTCTGCAACTCGACTCCATGA
AGTCGGAATCGCTAGTAATCGCAGATCAGCATTGCTGCGGTGAATACGTTCCCGGGCCT
TGTACACACCGCCCGTCACACCATGGGAGTTTGTTGCACCAGAAGCAGGTAGCTTAACC
TTCGGGAGGGCGCTGCCACGGGGTC

SH-3

Accession number: OR241335

GATGCCTTACAATGCAGTCGAACGGTAACAGGTCTTCGGACGCTGACGAGTGGCGAACG
GGTGAGTAATACATCGGAACGTGCCCAGTCGTGGGGGATAACTACTCGAAAGAGTAGCT
AATACCGCATACGATCTGAGGATGAAAGCGGGGGACCTTCGGGCCTCGCGCGATTGGAG
CGGCCGATGGCAGATTAGGTAGTTGGTGGGATAAAAGCTTACCAAGCCGACGATCTGTA
GCTGGTCTGAGAGGACGACCAGCCACACTGGGACTGAGACACGGCCCAGACTCCTACG
GGAGGCAGCAGTGGGGAATTTTGGACAATGGGCGAAAGCCTGATCCAGCAATGCCGCG
TGCAGGATGAAGGCCTTCGGGTTGTAAACTGCTTTTGTACGGAACGAAAAAGCTCCTTC
TAATACAGGGGGCCCATGACGGTACCGTAAGAATAAGCACCGGCTAACTACGTGCCAGC

AGCCGCGGTAATACGTAGGGTGCGAGCGTTAATCGGAATTACTGGGCGTAAAGCGTGCG
CAGGCGGTTATGTAAGACAGATGTGAAATCCCCGGGCTCAACCTGGGAACTGCATTTGT
GACTGCATGGCTAGAGTACGGTAGAGGGGGATGGAATTCCGCGTGTAGCAGTGAAATG
CGTAGATATGCGGAGGAACACCGATGGCGAAGGCAATCCCCTGGACCTGTACTGACGCT
CATGCACGAAAGCGTGGGGAGCAAACAGGATTAGATACCCTGGTAGTCCACGCCCTAA
ACGATGTCAACTGGTTGTTGGGAATTAGTTTTCTCAGTAACGAAGCTAACGCGTGAAGTT
GACCGCCTGGGGAGTACGGCCGCAAGGTTGAAACTCAAAGGAATTGACGGGGACCCGC
ACAAGCGGTGGATGATGTGGTTTAATTCGATGCAACGCGAAAAACCTTACCCACCTTTG
ACATGGCAGGAAGTTTCCAGAGATGGATTCGTGCTCGAAAGAGAACCTGCACACAGGTG
CTGCATGGCTGTCGTCAGCTCGTGTCGTGAGATGTTGGGTTAAGTCCCGCAACGAGCGC
AACCCTTGTCATTAGTTGCTACATTCAGTTGAGCACTCTAATGAGACTGCCGGTGACAAA
CCGGAGGAAGGTGGGGATGACGTCAAGTCCTCATGGCCCTTATAGGTGGGGCTACACAC
GTCATACAATGGCTGGTACAGAGGGTTGCCAACCCGCGAGGGGGAGCTAATCCCATAAA
ACCAGTCGTAGTCCGGATCGCAGTCTGCAACTCGACTGCGTGAAGTCGGAATCGCTAGT
AATCGCGGATCAGCATGCCGCGGTGAATACGTTCCCGGGTCTTGTACACACCGCCCGTC
ACACCATGGGAGCGGGTCTCGCCAGAAGTAGGTAGCCTAACCGCAAGGAGGGCGCTAC
CACGACG

SH-4

Accession number: OR241336

GGGGGAGGTCTACCATGCAAGTCGAGCGGATGACGGGAGCTTGCTCCTTGATTCAGCGG
CGGACGGGTGAGTAATGCCTAGGAATCTGCCTGGTAGTGGGGGACAACGTTTCGAAAGG
AACGCTAATACCGCATACGTCCTACGGGAGAAAGCAGGGGACCTTCGGGCCTTGCGCTA
TCAGATGAGCCTAGGTCGGATTAGCTAGTTGGTGGGGTAATGGCTCACCAAGGCGACGA
TCCGTAACTGGTCTGAGAGGATGATCAGTCACACTGGAACTGAGACACGGTCCAGACTC
CTACGGGAGGCAGCAGTGGGGAATATTGGACAATGGGCGAAAGCCTGATCCAGCCATG
CCGCGTGTGTGAAGAAGGTCTTCGGATTGTAAAGCACTTTAAGTTGGGAGGAAGGGCAG
TAAGTTAATACCTTGCTGTTTTGACGTTACCGACAGAATAAGCACCGGCTAACTCTGTGC
CAGCAGCCGCGGTAATACAGAGGGTGCAAGCGTTAATCGGAATTACTGGGCGTAAAGC
GCGCGTAGGTGGTTTGTTAAGTTGGATGTGAAAGCCCCGGGCTCAACCTGGGAACTGCA
TCCAAAACTGGCAAGCTAGAGTACGGTAGAGGGTGGTGGAATTTCCTGTGTAGCGGTGA
AATGCGTAGATATAGGAAGGAACACCAGTGGCGAAGGCGACCACCTGGACTGATACTG
ACACTGAGGTGCGAAAGCGTGGGGAGCAAACAGGATTAGATACCCTGGTAGTCCACGC
CGTAAACGATGTCAACTAGCCGTTGGAATCCTTGAGATTTTAGTGGCGCAGCTAACGCA
TTAAGTTGACCGCCTGGGGAGTACGGCCGCAAGGTTAAAACTCAAATGAATTGACGGGG
GCCCGCACAAGCGGTGGAGCATGTGGTTTAATTCGAAGCAACGCGAAGAACCTTACCAG
GCCTTGACATGCAGAGAACTTTCCAGAGATGGATTGGTGCCTTCGGGAACTCTGACACA
GGTGCTGCATGGCTGTCGTCAGCTCGTGTCGTGAGATGTTGGGTTAAGTCCCGTAACGA
GCGCAACCCTTGTCCTTAGTTACCAGCACGTTATGGTGGGCACTCTAAGGAGACTGCCG
GTGACAAACCGGAGGAAGGTGGGGATGACGTCAAGTCATCATGGCCCTTACGGCCTGG
GCTACACACGTGCTACAATGGTCGGTACAGAGGGTTGCCAAGCCGCGAGGTGGAGCTAA
TCTCACAAAACCGATCGTAGTCCGGATCGCAGTCTGCAACTCGACTGCGTGAAGTCGGA
ATCGCTAGTAATCGCGAATCAGAATGTCGCGGTGAATACGTTCCCGGGCCTTGTACACA
CCGCCCGTCACACCATGGGAGTGGGTTGCACCAGAAGTAGCTAGTCTAACCTTCGGGAG
GACGGTACCACGGGGATA

SH-5

Accession number: OR241337

GTGCGGAGGACTACCATGCAGTCGAACGGACCCTTCGGGGTTAGTGGCGGACGGGTGA
GTAACACGTGGGAACGTGCCTTTAGGTTCGGAATAGCTCCTGGAAACGGGTGGTAATGC
CGAATGTGCCCTTCGGGGGAAAGATTTATCGCCTTTAGAGCGGCCCGCGTCTGATTAGCT
AGTTGGTGAGGTAACGGCTCACCAAGGCGACGATCAGTAGCTGGTCTGAGAGGATGACC
AGCCACACTGGGACTGAGACACGGCCCAGACTCCTACGGGAGGCAGCAGTGGGGAATC
TTGCGCAATGGGCGAAAGCCTGACGCAGCCATGCCGCGTGAATGATGAAGGTCTTAGGA
TTGTAAAATTCTTTCACCGGGGACGATAATGACGGTACCCGGAGAAGAAGCCCCGGCTA
ACTTCGTGCCAGCAGCCGCGGTAATACGAAGGGGGCTAGCGTTGCTCGGAATTACTGGG
CGTAAAGGGCGCGTAGGCGGATCGTTAAGTCAGAGGTGAAATCCCAGGGCTCAACCCTG
GAACTGCCTTTGATACTGGCGATCTTGAGTATGAGAGAGGTATGTGGAACTCCGAGTGT
AGAGGTGAAATTCGTAGATATTCGGAAGAACACCAGTGGCGAAGGCGACATACTGGCT
CATTACTGACGCTGAGGCGCGAAAGCGTGGGGAGCAAACAGGATTAGATACCCTGGTA
GTCCACGCCGTAAACGATGATTGCTAGTTGTCGGGCTGCATGCAGTTCGGTGACGCAGC
TAACGCATTAAGCAATCCGCCTGGGGAGTACGGTCGCAAGATTAAAACTCAAAGGAATT
GACGGGGGCCCGCACAAGCGGTGGAGCATGTGGTTTAATTCGAAGCAACGCGCAGAAC
CTTACCACCTTTTGACATGCCTGGACCGCCACGGAGACGTGGCTTTCCCTTCGGGGACTA
GGACACAGGTGCTGCATGGCTGTCGTCAGCTCGTGTCGTGAGATGTTGGGTTAAGTCCC
GCAACGAGCGCAACCCTCGCCATTAGTTGCCATCATTTAGTTGGGAACTCTAATGGGAC
TGCCGGTGCTAAGCCGGAGGAAGGTGGGGATGACGTCAAGTCCTCATGGCCCTTACAGG
GTGGGCTACACACGTGCTACAATGGCAACTACAGAGGGTTAATCCTTAAAAGTTGTCTC
AGTTCGGATTGTCCTCTGCAACTCGAGGGCATGAAGTTGGAATCGCTAGTAATCGCGGA
TCAGCATGCCGCGGTGAATACGTTCCCGGGCCTTGTACACACCGCCCGTCACACCATGG
GAGTTGGTTCTACCCGAAGGCGGTGCGCTAACCAGCAATGGAGGCAGCCGACCACGTGG
TTC

SH-6

Accession number: OR241338

TGCGAGTGCTTACCTGCAGTCGAACGATGATCAGGAGCTTGCTCCTGTGATTAGTGGCG
AACGGGTGAGTAACACGTGAGTAACCTGCCCCTGACTCTGGGATAAGCGTTGGAAACGA
CGTCTAATACTGGATATGATCACTGGCCGCATGGTCTGGTGGTGGAAAGATTTTTGGTT
GGGGATGGACTCGCGGCCTATCAGCTTGTTGGTGAGGTAATGGCTCACCAAGGCGACGA
CGGGTAGCCGGCCTGAGAGGGTGACCGGCCACACTGGGACTGAGACACGGCCCAGACT
CCTACGGGAGGCAGCAGTGGGGAATATTGCACAATGGGCGAAAGCCTGATGCAGCAAC
GCCGCGTGAGGGATGACGGCCTTCGGGTTGTAAACCTCTTTTAGTAGGGAAGAAGCGAG
AGTGACGGTACCTGCAGAAAAAGCACCGGCTAACTACGTGCCAGCAGCCGCGGTAATA
CGTAGGGTGCAAGCGTTGTCCGGAATTATTGGGCGTAAAGAGCTCGTAGGCGGTTTGTC
GCGTCTGCTGTGAAATCCCGAGGCTCAACCTCGGGCTTGCAGTGGGTACGGGCAGACTA
GAGTGCGGTAGGGGAGATTGGAATTCCTGGTGTAGCGGTGGAATGCGCAGATATCAGG
AGGAACACCGATGGCGAAGGCAGATCTCTGGGCCGTAACTGACGCTGAGGAGCGAAAG
CGTGGGGAGCGAACAGGATTAGATACCCTGGTAGTCCACGCCGTAAACGTTGGGCGCTA
GATGTAGGGACCTTTCCACGGTTTCTGTGTCGTAGCTAACGCATTAAGCGCCCCGCCTGG
GGAGTACGGCCGCAAGGCTAAAACTCAAAGGAATTGACGGGGGCCCGCACAAGCGGCG
GAGCATGCGGATTAATTCGATGCAACGCGAAGAACCTTACCAAGGCTTGACATACACCG

GAAACGGCCAGAGATGGTCGCCCCCTTGTGGTCGGTGTACAGGTGGTGCATGGTTGTCG
TCAGCTCGTGTCGTGAGATGTTGGGTTAAGTCCCGCAACGAGCGCAACCCTCGTTCTATG
TTGCCAGCGCGTTATGGCGGGGACTCATAGGAGACTGCCGGGGTCAACTCGGAGGAAG
GTGGGGATGACGTCAAATCATCATGCCCCTTATGTCTTGGGCTTCACGCATGCTACAATG
GCCGGTACAAAGGGCTGCGATACCGTAAGGTGGAGCGAATCCCAAAAAGCCGGTCTCA
GTTCGGATTGAGGTCTGCAACTCGACCTCATGAAGTCGGAGTCGCTAGTAATCGCAGAT
CAGCAACGCTGCGGTGAATACGTTCCCGGGCCTTGTACACACCGCCCGTCAAGTCATGA
AAGTCGGTAACACCCGAAGCCGGTGGCCTAACCCTTGTGGGAGGAGCCGTCGAAGGGC
ATT

SH-7

Accession number: OR241339

TGCGGGGTGCTTACCATGCAAGTCGAACGGTGAAGCAGGAGCTTGCTCTTGTGGATCAG
TGGCGAACGGGTGAGTAACACGTGAGCAACCTGCCCCTGACTCTGGGATAAGCGCTGGA
AACGGCGTCTAATACTGGATATGTGACGTGACCGCATGGTCTGCGTTTGGAAAGATTTTT
CGGTTGGGGATGGGCTCGCGGCCTATCAGCTTGTTGGTGAGGTAATGGCTCACCAAGGC
GTCGACGGGTAGCCGGCCTGAGAGGGTGACCGGCCACACTGGGACTGAGACACGGCCC
AGACTCCTACGGGAGGCAGCAGTGGGGAATATTGCACAATGGGCGAAAGCCTGATGCA
GCAACGCCGCGTGAGGGATGACGGCCTTCGGGTTGTAAACCTCTTTTAGCAGGGAAGAA
GCGAAAGTGACGGTACCTGCAGAAAAAGCGCCGGCTAACTACGTGCCAGCAGCCGCGG
TAATACGTAGGGCGCAAGCGTTATCCGGAATTATTGGGCGTAAAGAGCTCGTAGGCGGT
TTGTCGCGTCTGCTGTGAAATCCCGAGGCTCAACCTCGGGCCTGCAGTGGGTACGGGCA
GACTAGAGTGCGGTAGGGGAGATTGGAATTCCTGGTGTAGCGGTGGAATGCGCAGATAT
CAGGAGGAACACCGATGGCGAAGGCAGATCTCTGGGCCGTAACTGACGCTGAGGAGCG
AAAGGGTGGGGAGCAAACAGGCTTAGATACCCTGGTAGTCCACCCCGTAAACGTTGGG
AACTAGTTGTGGGGTCCATTCCACGGATTCCGTGACGCAGCTAACGCATTAAGTTCCCCG
CCTGGGGAGTACGGCCGCAAGGCTAAAACTCAAAGGAATTGACGGGGACCCGCACAAG
CGGCGGAGCATGCGGATTAATTCGATGCAACGCGAAGAACCTTACCAAGGCTTGACATA
TACGAGAACGGGCCAGAAATGGTCAACTCTTTGGACACTCGTAAACAGGTGGTGCATGG
TTGTCGTCAGCTCGTGTCGTGAGATGTTGGGTTAAGTCCCGCAACGAGCGCAACCCTCGT
TCTATGTTGCCAGCACGTAATGGTGGGAACTCATGGGATACTGCCGGGGTCAACTCGGA
GGAAGGTGGGGATGACGTCAAATCATCATGCCCCTTATGTCTTGGGCTTCACGCATGCT
ACAATGGCCGGTACAAAGGGCTGCAATACCGTGAGGTGGAGCGAATCCCAAAAAGCCG
GTCCCAGTTCGGATTGAGGTCTGCAACTCGACCTCATGAAGTCGGAGTCGCTAGTAATC
GCAGATCAGCAACGCTGCGGTGAATACGTTCCCGGGTCTTGTACACACCGCCCGTCAAG
TCATGAAAGTCGGTAACACCTGAAGCCGGTGGCCTAACCCTTGTGGAGGGAGCCGTCGA
AGGGTAT

S1.3. Supplementary Tables

Supplementary Table S1. Data of PHE degradation ratio

	8 h	16 h	24 h	32 h	40 h	48 h	56 h	64 h	72 h
HJ-SH	0.128±0.009	0.24±0.015	0.472±0.021	0.664±0.032	0.919±0.04	0.924±0.052	0.931±0.035	0.945±0.022	0.981±0.015
HJ-7	0.045±0.005	0.15±0.009	0.328±0.026	0.432±0.065	0.464±0.052	0.859±0.045	0.895±0.055	0.899±0.035	0.938±0.017
HJ-SH1(-)	0.01±0.009	0.021±0.013	0.023±0.021	0.027±0.026	0.059±0.025	0.2±0.019	0.121±0.026	0.237±0.021	0.331±0.039
HJ-SH2(-)	0.073±0.019	0.084±0.027	0.09±0.029	0.104±0.019	0.134±0.031	0.253±0.029	0.295±0.025	0.342±0.036	0.292±0.047
HJ-SH3(-)	0.031±0.023	0.024±0.019	0.135±0.027	0.115±0.031	0.141±0.012	0.216±0.009	0.237±0.007	0.243±0.029	0.387±0.037
HJ-SH4(-)	0.004±0.002	0.016±0.013	0.021±0.02	0.029±0.023	0.036±0.025	0.156±0.048	0.181±0.036	0.181±0.028	0.225±0.014
HJ-SH5(-)	0.01±0.001	0.011±0.005	0.057±0.014	0.07±0.021	0.069±0.028	0.11±0.04	0.13±0.026	0.254±0.022	0.299±0.04
HJ-SH6(-)	0.024±0.021	0.036±0.035	0.058±0.032	0.077±0.026	0.101±0.036	0.124±0.041	0.149±0.043	0.307±0.045	0.596±0.037
HJ-SH7(-)	0.002±0.001	0.04±0.019	0.075±0.007	0.127±0.015	0.113±0.029	0.144±0.03	0.194±0.033	0.228±0.047	0.354±0.02
HJ-1	0.009±0.015	0.011±0.025	0.023±0.023	0.044±0.036	0.053±0.049	0.058±0.032	0.115±0.029	0.12±0.031	0.155±0.041
HJ-2	0.001±0.001	0.018±0.017	0.023±0.024	0.041±0.014	0.053±0.029	0.056±0.037	0.084±0.028	0.084±0.03	0.122±0.039

HJ-3	0.002±0.001	0.012±0.021	0.028±0.018	0.052±0.034	0.078±0.025	0.127±0.036	0.123±0.033	0.132±0.028	0.161±0.019
HJ-4	0.020±0.002	0.059±0.023	0.041±0.036	0.066±0.025	0.066±0.026	0.068±0.029	0.096±0.027	0.184±0.037	0.378±0.041
HJ-5	0.014±0.0021	0.01±0.031	0.035±0.039	0.049±0.037	0.067±0.029	0.092±0.048	0.103±0.027	0.076±0.025	0.131±0.032
HJ-6	0.04±0.017	0.058±0.019	0.043±0.029	0.047±0.037	0.067±0.036	0.076±0.025	0.092±0.032	0.127±0.039	0.076±0.019
HJ-7	0.007±0.001	0.011±0.025	0.023±0.029	0.048±0.03	0.043±0.032	0.093±0.033	0.118±0.023	0.153±0.037	0.214±0.02

Supplementary Table S2. Data of OD_{600}

	8 h	16 h	24 h	32 h	40 h	48 h	56 h	64 h	72 h
HJ-SH	0.258±0.015	0.441±0.035	0.82±0.049	1.123±0.055	1.778±0.062	1.976±0.098	2.625±0.102	2.888±0.082	2.455±0.119
HJ-7	0.198±0.01	0.401±0.037	0.759±0.031	0.883±0.051	1.102±0.06	1.795±0.059	2.197±0.098	2.757±0.088	2.45±0.095
HJ-SH1(-)	0.567±0.033	0.609±0.027	0.617±0.025	1.075±0.037	1.525±0.046	1.516±0.047	1.782±0.066	2.075±0.104	1.536±0.099
HJ-SH2(-)	0.393±0.026	0.478±0.014	0.504±0.036	1.046±0.07	1.327±0.097	1.512±0.087	1.93±0.102	2.07±0.075	1.527±0.07
HJ-SH3(-)	0.373±0.014	0.356±0.021	0.942±0.058	1.342±0.07	1.547±0.06	1.939±0.09	2.035±0.105	2.248±0.112	1.839±0.07
HJ-SH4(-)	0.22±0.019	0.273±0.021	0.893±0.06	1.308±0.068	1.456±0.069	1.602±0.079	1.892±0.057	1.703±0.07	1.284±0.099

HJ-SH5(-)	0.871±0.039	0.972±0.078	1.272±0.079	1.558±0.082	1.685±0.059	1.716±0.036	1.787±0.069	1.816±0.108	1.684±0.079
HJ-SH6(-)	0.882±0.048	0.882±0.039	1.139±0.089	1.777±0.088	1.803±0.098	1.818±0.056	1.995±0.103	2.023±0.112	1.806±0.069
HJ-SH7(-)	0.95±0.033	1.057±0.05	1.282±0.057	1.788±0.088	1.812±0.07	2.085±0.102	2.262±0.124	2.264±0.097	2.211±0.087
HJ-1	0.258±0.035	0.269±0.037	0.488±0.026	0.689±0.045	0.723±0.069	0.856±0.053	0.964±0.044	0.825±0.052	0.778±0.07
HJ-2	0.385±0.026	0.789±0.033	0.857±0.07	0.889±0.019	0.879±0.052	0.949±0.064	0.984±0.078	0.895±0.099	0.896±0.078
HJ-3	0.457±0.019	0.694±0.021	0.878±0.037	0.932±0.059	1.096±0.065	1.079±0.066	1.295±0.075	1.176±0.026	1.115±0.037
HJ-4	0.458±0.026	0.596±0.037	0.789±0.048	0.965±0.051	0.956±0.047	1.198±0.046	1.287±0.06	1.359±0.12	1.299±0.052
HJ-5	0.296±0.021	0.479±0.013	0.525±0.04	0.726±0.046	0.969±0.069	0.957±0.098	0.969±0.079	1.017±0.065	0.911±0.042
HJ-6	0.255±0.014	0.268±0.025	0.487±0.014	0.579±0.05	0.596±0.055	0.688±0.078	0.956±0.106	0.969±0.099	0.823±0.053
HJ-7	0.225±0.013	0.397±0.026	0.461±0.037	0.467±0.035	0.523±0.037	0.669±0.059	0.796±0.043	0.709±0.012	0.708±0.07

Supplementary Table S3. Data of relative PHE degradation rate

	8 h	16 h	24 h	32 h	40 h	48 h	56 h	64 h	72 h
HJ-SH	0.498±0.015	0.545±0.009	0.576±0.02	0.591±0.001	0.517±0.006	0.468±0.026	0.355±0.013	0.327±0.002	0.4±0.015
HJ-7	0.231±0.031	0.379±0.053	0.431±0.016	0.487±0.048	0.422±0.052	0.478±0.01	0.408±0.022	0.326±0.014	0.383±0.012

HJ-SH1(-)	0.036±0.008	0.041±0.007	0.048±0.006	0.063±0.009	0.074±0.011	0.067±0.012	0.119±0.016	0.146±0.026	0.199±0.025
HJ-SH2(-)	0.003±0.001	0.023±0.003	0.027±0.009	0.046±0.017	0.06±0.024	0.059±0.022	0.085±0.026	0.094±0.019	0.136±0.017
HJ-SH3(-)	0.004±0.003	0.017±0.005	0.032±0.014	0.055±0.017	0.071±0.015	0.118±0.022	0.095±0.026	0.112±0.028	0.144±0.017
HJ-SH4(-)	0.043±0.005	0.099±0.005	0.051±0.015	0.068±0.017	0.069±0.017	0.057±0.02	0.075±0.014	0.135±0.017	0.291±0.025
HJ-SH5(-)	0.047±0.01	0.021±0.02	0.067±0.02	0.068±0.021	0.07±0.015	0.096±0.009	0.106±0.017	0.075±0.027	0.144±0.02
HJ-SH6(-)	0.157±0.012	0.217±0.01	0.087±0.022	0.081±0.022	0.113±0.025	0.11±0.014	0.096±0.026	0.131±0.028	0.092±0.017
HJ-SH7(-)	0.031±0.008	0.028±0.012	0.049±0.014	0.103±0.022	0.083±0.017	0.139±0.026	0.148±0.031	0.216±0.024	0.303±0.022
HJ-1	0.036±0.007	0.041±0.012	0.048±0.045	0.063±0.04	0.074±0.036	0.067±0.03	0.119±0.033	0.146±0.03	0.199±0.029
HJ-2	0.003±0.001	0.023±0.026	0.027±0.014	0.046±0.037	0.06±0.028	0.059±0.029	0.085±0.018	0.094±0.015	0.136±0.025
HJ-3	0.004±0.002	0.017±0.028	0.032±0.024	0.055±0.029	0.071±0.019	0.118±0.027	0.095±0.022	0.112±0.055	0.144±0.026
HJ-4	0.043±0.035	0.099±0.031	0.051±0.038	0.068±0.024	0.069±0.028	0.057±0.032	0.075±0.023	0.135±0.015	0.291±0.039
HJ-5	0.047±0.027	0.021±0.036	0.067±0.041	0.068±0.041	0.07±0.021	0.096±0.024	0.106±0.017	0.075±0.019	0.144±0.038
HJ-6	0.157±0.063	0.217±0.038	0.087±0.107	0.081±0.037	0.113±0.033	0.11±0.016	0.096±0.015	0.131±0.02	0.092±0.018
HJ-7	0.031±0.022	0.028±0.019	0.049±0.026	0.103±0.043	0.083±0.043	0.139±0.028	0.148±0.027	0.216±0.054	0.303±0.014

Supplementary Table S4. Data of PHE tolerance experiment

	12 h	24 h	36 h	48 h	60 h	72 h	84 h	96 h	108 h	120h
Degradation ratio										
1 g/L	0.104±0.01	0.313±0.025	0.428±0.021	0.446±0.027	0.513±0.037	0.668±0.032	0.755±0.027	0.877±0.025	0.911±0.028	0.926±0.037
2 g/L	0.079±0.011	0.201±0.014	0.328±0.021	0.309±0.02	0.396±0.023	0.409±0.022	0.596±0.031	0.779±0.023	0.89±0.039	0.9±0.032
3 g/L	0.056±0.018	0.118±0.022	0.158±0.025	0.208±0.011	0.338±0.017	0.349±0.021	0.394±0.036	0.502±0.022	0.518±0.04	0.509±0.022
4 g/L	0.012±0.004	0.051±0.011	0.098±0.013	0.103±0.02	0.15±0.015	0.198±0.014	0.208±0.012	0.27±0.029	0.307±0.031	0.307±0.022
4.5 g/L	0.009±0.005	0.058±0.02	0.076±0.011	0.088±0.016	0.096±0.018	0.158±0.022	0.169±0.027	0.159±0.031	0.18±0.021	0.181±0.033
OD_{600}										
1 g/L	0.189±0.103	0.371±0.151	0.903±0.211	1.479±0.269	1.828±0.111	2.102±0.123	2.302±0.197	2.311±0.152	2.41±0.275	2.568±0.166
2 g/L	0.13±0.112	0.291±0.136	0.749±0.106	1.261±0.196	1.484±0.123	1.962±0.219	2.25±0.209	2.411±0.127	2.509±0.293	2.432±0.118
3 g/L	0.078±0.177	0.258±0.119	0.666±0.253	1.128±0.112	1.313±0.169	1.503±0.109	1.589±0.155	1.647±0.121	1.698±0.196	1.728±0.215
4 g/L	0.059±0.12	0.28±0.111	0.584±0.125	0.743±0.203	0.824±0.154	0.976±0.142	1.059±0.116	1.093±0.293	0.809±0.209	0.801±0.124
4.5 g/L	0.049±0.113	0.124±0.091	0.223±0.109	0.276±0.126	0.371±0.178	0.517±0.115	0.586±0.097	0.669±0.113	0.575±0.109	0.484±0.127

Supplementary Table S5. Data of degradation ratio

	12 h	24 h	36 h	48 h	60 h	72 h	84 h	96 h	108 h	120h
Biphenyl	0.198±0.026	0.251±0.02	0.396±0.026	0.498±0.026	0.666±0.036	0.737±0.05	0.87±0.04	0.898±0.056	0.921±0.069	0.931±0.042
Anthracene	0.109±0.029	0.18±0.013	0.276±0.01	0.38±0.026	0.6±0.038	0.71±0.034	0.78±0.042	0.805±0.051	0.896±0.046	0.916±0.04
Hexadecane	0.058±0.018	0.097±0.026	0.152±0.018	0.18±0.033	0.209±0.04	0.36±0.041	0.476±0.033	0.549±0.058	0.66±0.054	0.701±0.046
